# Different Involvement of Band 3 in Red Cell Deformability and Osmotic Fragility—A Comparative GP.Mur Erythrocyte Study

**DOI:** 10.3390/cells10123369

**Published:** 2021-11-30

**Authors:** Mei-Shin Kuo, Cheng-Hsi Chuang, Han-Chih Cheng, Hui-Ru Lin, Jong-Shyan Wang, Kate Hsu

**Affiliations:** 1 The Department of Laboratory Medicine, Taitung MacKay Memorial Hospital, Taitung 950, Taiwan; a1446@ttms.mmh.org.tw (M.-S.K.); ah118@ttms.mmh.org.tw (H.-C.C.); 2 Department of mechanical and mechatronic engineering, National Taiwan Ocean University, Keelung 202, Taiwan; chchuang20@mail.ntou.edu.tw; 3 The Laboratory of Immunogenetics, Department of Medical Research, MacKay Memorial Hospital, Tamsui, New Taipei City 251, Taiwan; linmaegan@gmail.com; 4 Healthy Aging Research Center, Graduate Institute of Rehabilitation Science, Chang Gung University, Taoyuan 333, Taiwan; s5492@mail.cgu.edu.tw; 5 Department of Physical Medicine and Rehabilitation, Keelung Chang Gung Memorial Hospital, Keelung 204, Taiwan; 6 Research Center for Chinese Herbal Medicine, College of Human Ecology, Chang Gung University of Science and Technology, Taoyuan 333, Taiwan; 7 Department of Nursing, MacKay Junior College of Medicine, Nursing, and Management, New Taipei City 252, Taiwan; 8 Institute of Biomedical Sciences, MacKay Medical College, New Taipei City 252, Taiwan

**Keywords:** erythrocyte (red blood cells), GP.Mur (Miltenberger subtype III), band 3, deformability, osmotic fragility, AQP1 (aquaporin-1), membrane cholesterol, microcytosis, mean corpuscular volume (MCV)

## Abstract

GP.Mur is a clinically important red blood cell (RBC) phenotype in Southeast Asia. The molecular entity of GP.Mur is glycophorin B-A-B hybrid protein that promotes band 3 expression and band 3–AQP1 interaction, and alters the organization of band 3 complexes with Rh/RhAG complexes. GP.Mur+ RBCs are more resistant to osmotic stress. To explore whether GP.Mur+ RBCs could be structurally more resilient, we compared deformability and osmotic fragility of fresh RBCs from 145 adults without major illness (47% GP.Mur). We also evaluated potential impacts of cellular and lipid factors on RBC deformability and osmotic resistivity. Contrary to our anticipation, these two physical properties were independent from each other based on multivariate regression analyses. GP.Mur+ RBCs were less deformable than non-GP.Mur RBCs. We also unexpectedly found 25% microcytosis in GP.Mur+ female subjects (10/40). Both microcytosis and membrane cholesterol reduced deformability, but the latter was only observed in non-GP.Mur and not GP.Mur+ normocytes. The osmotic fragility of erythrocytes was not affected by microcytosis; instead, larger mean corpuscular volume (MCV) increased the chances of hypotonic burst. From comparison with GP.Mur+ RBCs, higher band 3 expression strengthened the structure of RBC membrane and submembranous cytoskeletal networks and thereby reduced cell deformability; stronger band 3–AQP1 interaction additionally supported osmotic resistance. Thus, red cell deformability and osmotic resistivity involve distinct structural–functional roles of band 3.

## 1. Introduction

GP.Mur is a special red cell type with 1–10% prevalence in Southeast Asia [1,2]. GP stands for glycoprotein. Mur is the name of the main antigen in the Miltenberger series of the MNS blood group system that could trigger allogenic reactivities and acute hemolysis in the event of blood type incompatibility (i.e., during mismatched transfusion) [3,4]. GP.Mur protein, a variant of glycophorin B, functions like homologous glycophorin A in supporting band 3 complex formation in the endoplasmic reticulum [5,6,7]. Band 3, the most abundant membrane protein in red cells, is a bidirectional Cl^-^/HCO_3_^–^ transporter essential for physiologic CO_2_/HCO_3_^–^ homeostasis [8,9,10]. On the red cell membrane, GP.Mur oligomerizes with glycophorin A and band 3, and supports band 3 expression [5]. GP.Mur+ band 3 complexes are unique in (i) stronger association with AQP1 [11] and (ii) differential structural organization with Rh/RhAG complexes [12,13]. AQP1 is a gated water channel that functions in response to changes of osmolarity [14,15]. GP.Mur+ RBCs are structurally more resistant to hypotonically induced rupture than non-GP.Mur RBCs [5]. For this reason, we hypothesized that GP.Mur expression could also influence membrane resilience and affect red cell deformability.

Both deformability and osmotic fragility are physical properties applied to diagnostics of abnormal or unstable erythrocytes, such as spherocytosis and hemolytic anemia. Deformability measures the extensibility of an erythrocyte upon mechanical stress. Red cell deformability is influenced by (1) surface area (SA)-to-volume (V) ratio of a cell, (2) intracellular viscosity, and (3) membrane viscoelasticity [16]. The intracellular viscosity of an erythrocyte is primarily reflected by the mean corpuscular hemoglobin concentration (MCHC) [17]. The membrane viscoelasticity of an erythrocyte is determined by the strengths of horizontal linkages (cell membrane and submembranous cytoskeleton) and of vertical linkages (mainly band 3-ankyrin complexes and band 3-adducin complexes) [16,18,19,20,21]. These band 3 complexes on the RBC membrane serve as bollards anchoring on submembranous vertices of cytoskeletal hexagons of triangles [22].

Differing from deformability, osmotic fragility measures cell membrane extensibility upon hypotonic stress. Because osmotic fragility is measured by changing tonicity, this method is sensitive to movements of ions and water across the red cell membrane. The direction of ion (or water) flux through band 3 (or AQP1) follows the concentration gradients of individual ions (or water) if no cellular or molecular regulation is imposed. AQP1 and band 3 interact transiently on the surface of red cells; their protein–protein interaction disappears when red cells swell in a hypotonic milieu [11]. Intriguingly, the interaction between AQP1 and band 3 on the GP.Mur+ cell membrane is stronger [11]. How their interaction could affect RBC stability and resistivity to osmotic stress is largely unclear.

## 2. Materials and Methods

This study compared biophysical properties of GP.Mur+ and non-GP.Mur erythrocyte samples that were collected from nondiseased adult subjects. People who were interested in participating in the study were first asked to complete a questionnaire on their health status. Based on their answers to the questionnaire, those with severe cardiovascular, diabetic, hematological, or other major catastrophic diseases or conditions defined by the Taiwan National Health Insurance (TW-NHI) criteria [23] were excluded from the study. The study protocol was approved by the MacKay Memorial Hospital (MMH) Institutional Review Board (MMH-IRB registration: 11MMHIS038). All recruited subjects provided written consents.

### 2.1. Blood Sample Processing

Peripheral blood was collected in an EDTA tube, a serum gel tube, and an ACD tube. A portion of the blood sample collected in the ACD tube was washed, fractionated, and aliquoted for RBC storage in a −80 °C freezer for later analyses. Fresh blood samples were analyzed for complete blood count (CBC), routine RBC typing, serum lipid analyses, osmotic fragility tests, and red cell deformability. Frozen aliquots of washed RBCs were subjected to membrane cholesterol analyses.

### 2.2. GP.Mur Phenotyping

GP.Mur blood type was serologically screened using anti-Mi^a^ and anti-Mur antisera. The serologically positive samples were then verified by *GYP.Mur*-typing PCR as described in [24].

### 2.3. Quantitation of Red Cell Membrane Cholesterol

Membrane cholesterol was quantitated from frozen packed RBC samples using the Amplex^TM^ Red Cholesterol Assay kit (Invitrogen, Carlsbad, CA, USA). Briefly, a vial of 200 μL frozen RBC sample was centrifuged to remove the storage buffer, followed by PBS wash. All processes from this point on were on ice. The RBC pellet was hypotonically lysed with 1 mL of ice-cold 10 mM Tris buffer, pH 8.0, followed by centrifuge at 14,000× *g* and 4 °C to obtain the ghost (membrane) pellet. The ghost pellet was vortex-mixed with 100 μL of 1% Triton X-100 in PBS. The membrane cholesterol content in this detergent-lysed sample was quantified using the Amplex^TM^ kit, according to the company’s instruction manual.

### 2.4. RBC Deformability Test

RBC deformability was measured using the Rheoscan-D ektacytometer (RheoMeditech, Seoul, Korea). The test analyzed fresh blood samples that were collected in ACD tubes. Before loaded onto Rheoscan-D, 6 μL of washed RBCs was diluted in 600 μL of polyvinylpyrrolidone phosphate buffered solution (5.5% polyvinylpyrrolidone; 300 mOsm/L). The loaded sample was subjected to rotational speeds with increasing shear stresses ranging from 0.3 to 30 Pa at 37 °C. Images of the stress-induced, deformed RBCs were captured by laser diffraction. The elongation index (EI) of an individual RBC at a given shear stress is defined as (A − B)/(A + B), where A is the major (or longest) axial length and B the minor (or shortest) axial length of the imaged RBC. Rheoscan-D also calculated EI_max_ (derived maximal EI) and SS_1/2_ (shear stress capable of triggering 50% EI_max_) based on the experimental EI profile [25,26].

### 2.5. Osmotic Fragility Test

Five microliters of fresh whole blood was each added to 0.5 mL of serially diluted NaCl solutions (concentrations ranging from 1% to 0.1% NaCl in ddH_2_O), followed by gentle mix and 30 min incubation at room temperature. They were then centrifuged for 5 min to pellet unruptured RBCs. Interpretation of the osmotic fragility test results should begin from the highest to the lowest concentrations of NaCl over a white board or a white background. The beginning of hemolysis was determined by visually comparing the test samples with a negative control for the first occurrence of faint pink-reddish color in the supernatant after centrifuge. The test recorded %NaCl at which hemolysis began and ended for each sample. The former was the concentration of NaCl at which pink color started to show in the supernatant; the latter was the concentration of NaCl at which the RBC pellet was no longer visible.

### 2.6. Statistics

The independent *t*-test was used to compare the means of two groups. Multivariate linear regression was used to evaluate the influences of potential factors (independent variables) on RBC deformability or on osmotic resistivity (dependent variable); *p* < 0.05 was deemed statistically significant. Statistics and plotting were performed using SPSS version 26 (IBM) and OriginPro 2020 software, respectively.

## 3. Results

This study examined the influence of GP.Mur phenotype on RBC deformability and osmotic resistibility. Among the 145 adult subjects recruited (59 men and 86 women), 13 had microcytosis with mean corpuscular volume (MCV) < 80 μL (Table 1). Intriguingly, 10 out of the 13 microcytic cases were GP.Mur+ women, two were GP.Mur+ men, and one was a non-GP.Mur man. This high propensity of microcytosis in GP.Mur+ women (25%, 10/40) suggests a likelihood of gene linkage. Microcytosis was identified by CBC tests: it significantly reduced MCV, hemoglobin (Hb) content, % hematocrit, mean corpuscular Hb concentration (MCHC), and mean corpuscular Hb (MCH), while increased red cell distribution width (RDW) and RBC count (Table 1). The latter is to physiologically compensate for microcytic hypochromia. From separate analyses of microcytes and normocytes (normal-sized RBCs), we did not find GP.Mur phenotype to influence any CBC parameters of the normocytes (Table 1).

### 3.1. GP.Mur+ RBCs Were More Osmotically Resistant and Less Deformable

**Figure 1:**
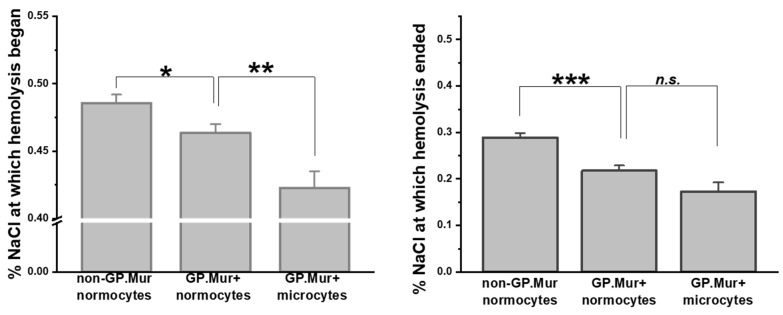
GP.Mur+ RBCs exhibited superior resistance to osmotic stress. Osmotic fragility tests showed that GP.Mur+ RBCs began and completed hemolysis at more hypotonic concentrations than non-GP.Mur cells. GP.Mur+ microcytes began hemolysis at even more hypotonic concentrations than GP.Mur+ normocytes, but GP.Mur+ microcytes and normocytes were not different in the %NaCl at which hemolysis terminated. Data are presented in mean ± SEM; * p < 0.05; ** p < 0.01; *** p < 0.001; *n.s*., not significant.

Compared to the previous report on osmotic resistivity using a small number of GP.Mur+ subjects [5], here we confirmed with a significantly larger sample size that GP.Mur+ normocytes were more resistant to osmotic stress than non-GP.Mur (Figure 1 and Table 2). Their difference was most pronounced in the concentrations of NaCl at which hemolysis terminated (*** p < 0.001): 0.218 ± 0.070% (GP.Mur) versus 0.288 ± 0.067% (non-GP.Mur). Notably, GP.Mur+ microcytes were able to tolerate more hypotonic conditions than GP.Mur+ normocytes in the beginning (Figure 1, similar %NaCl at which hemolysis began).

**Figure 2:**
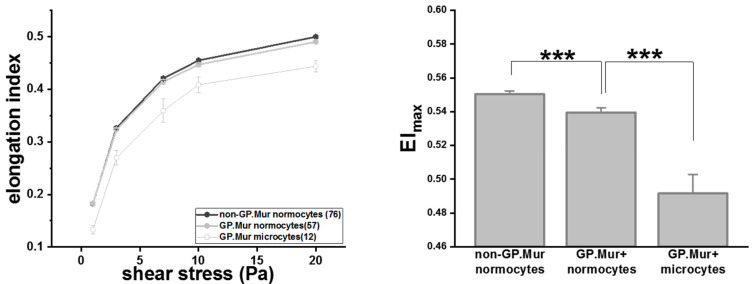
GP.Mur+ RBCs were less deformable than non-GP.Mur RBCs and GP.Mur+ microcytes were least deformable compared to normocytes. Left: The elongation indices increased as the given shear stresses increased. The number of subjects tested per group is indicated in the parentheses in the legend box. Right: All three groups were significantly different from one another; *** p < 0.001.

RBC deformability tests started with a small shear stress and increased gradually. At shear stress < 20 Pa, the degrees of deformability, as measured in elongation index (EI), were similar between GP.Mur+ and non-GP.Mur normocytes (Table 2). GP.Mur+ normocytes became slightly but significantly less deformable than non-GP.Mur normocytes when challenged with 20 Pa or higher shear stress (Table 2 and Figure 2). Noticeably, the elongation indices of GP.Mur+ microcytes were much lower than that of GP.Mur+ or non-GP.Mur normocytes throughout all levels of the shear stresses tested (Figure 2 and Table 2). This indicates that the morphological differences between microcytes and normocytes influenced RBC deformability much more than GP.Mur type did.

### 3.2. The Rigidifying Effect of Membrane Cholesterol on Deformability Was Absent in GP.Mur+ RBCs

Despite that both deformability and osmotic resistibility reflect the resilience of red cell membrane, they are distinct physical methods. Using multivariate regression analyses, we thus probed into how they could each be affected by cellular or lipid factors. We assigned the most pronounced marker of deformability, EI_max_, as the dependent variable in the multivariate regression, and first assessed the effects of gender, GP.Mur type, microcytosis, and the two parameters of osmotic fragility (namely, %NaCl at which hemolysis began and ended). RBC deformability was independent from the two parameters of osmotic fragility in this model (Table 3). Though male and female differed slightly in CBC, this gender difference did not influence red cell deformability (Table 3). Among these independent variables tested, only microcytosis and GP.Mur phenotype affected EI_max_ significantly (Table 3).

We next grouped subjects based on their GP.Mur and microcytic status, and examined the potential impacts of cellular (CBC) and lipid factors on red cell deformability. We found that the impact of MCV to EI_max_ was limited to GP.Mur+ microcytic samples (Table 4) or to microcytic cases in general. The deformability of normocytes was generally not affected by cell volume differences (Table 4). We did not find MCHC or RDW to significantly affect deformability when analyzing in the three separate groups (non-GP.Mur normocytic, GP.Mur normocytic, and GP.Mur microcytic groups), though it has been reported that increasing deformability could be associated with lower MCHC and higher MCV in healthy, middle-aged women [27]. MCHC reflects the intracellular viscosity [17]. Thus, intracellular viscosity would not be the major cause of different deformability between GP.Mur and non-GP.Mur (Figure 2 and Table 2). If we computed all data without grouping or excluding microcytic cases, RDW would be the only CBC parameter with significant contribution to cell deformability (data not shown). However, because many microcytic cases were linked to GP.Mur phenotype in this cohort (Table 1), the analysis without grouping (as in Table 4) would result in biased interpretation of the data.

**Figure 3:**
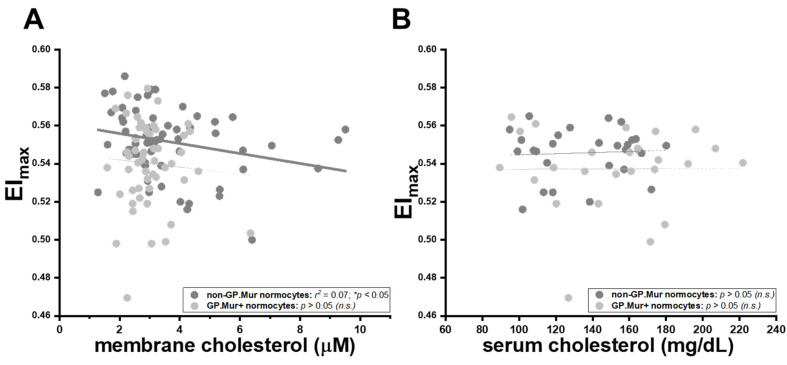
The rigidifying effects of membrane cholesterol on red cell deformability were significant in non-GP.Mur RBCs but not in GP.Mur+ RBCs. Each dot represents the data of a subject with normal RBC sizes. The impacts of (**A**) membrane cholesterol and (**B**) serum cholesterol on maximal elongation indices (EI_max_) are presented. Significant linear fitting (* p < 0.05) is presented using a solid line, poor or failed linear fitting (*p* > 0.05) in thin dashed lines. *n.s*., not significant.

Intriguingly, the well-established inverse relation between membrane cholesterol content and erythrocyte deformability was only observed in non-GP.Mur normocytes, and not in GP.Mur+ normocytes or microcytes (Table 4) [28,29]. Insertion of cholesterol into the cellular membrane makes the membrane less fluidic or bendable [28,29,30]. For non-GP.Mur RBCs, increasing membrane cholesterol rigidified red cell membrane and reduced cell deformability (non-GP.Mur in Figure 3A: *r* = 0.26, * p < 0.05). However, membrane cholesterol did not affect the deformability of GP.Mur+ RBCs (Figure 3A: poor linear fitting). As a control, serum cholesterol exerted no influence on RBC deformability (Figure 3B).

We also observed lower membrane cholesterol levels in GP.Mur+ than in non-GP.Mur RBCs (Table 5). Serum cholesterol levels between GP.Mur+ and non-GP.Mur subjects were not significantly different (Table 5). It is largely unclear what drives cholesterol exchange between the plasma and the RBC membrane [31,32,33,34]. Based on the data in Figure 3, GP.Mur/higher band 3 expression influenced deformability more than membrane cholesterol did; it also limited the content of membrane cholesterol on the red cells through an unknown mechanism.

### 3.3. Superior Osmotic Resistance of GP.Mur+ RBCs Likely Due to Stronger AQP1-Band 3 Binding

By multivariate regression analysis, we also examined the potential factors that could affect the osmotic resistance of RBCs, including GP.Mur type, microcytosis, MCV, RDW, MCHC, and EI_max_. We again found that RBC deformability and osmotic resistivity were independent from each other (Table 3 and Table 6). GP.Mur was the most significant contributor to osmotic resistibility in this cohort that comprised 47% GP.Mur+ subjects (Table 6). Microcytosis did not affect osmotic resistibility (Table 6), though it significantly reduced RBC deformability (Table 3). On the other hand, even after controlling for microcytosis in the multivariate regression model for osmotic fragility, increasing MCV significantly increased osmotic fragility (Table 6: standardized coefficient β 0.392, ** p < 0.01). As band 3 and AQP1 interact more strongly on GP.Mur+ RBCs and the degree of their interaction depends on tonicity [11], these data suggest that water flux in GP.Mur+ RBCs could be more strictly regulated to prevent hypotonic burst. Thus, water permeation through aquaporin and regulation of aquaporin activities could be critical determinants for cell volume and osmotic fragility of erythrocytes.

## 4. Discussion

**Figure 4:**
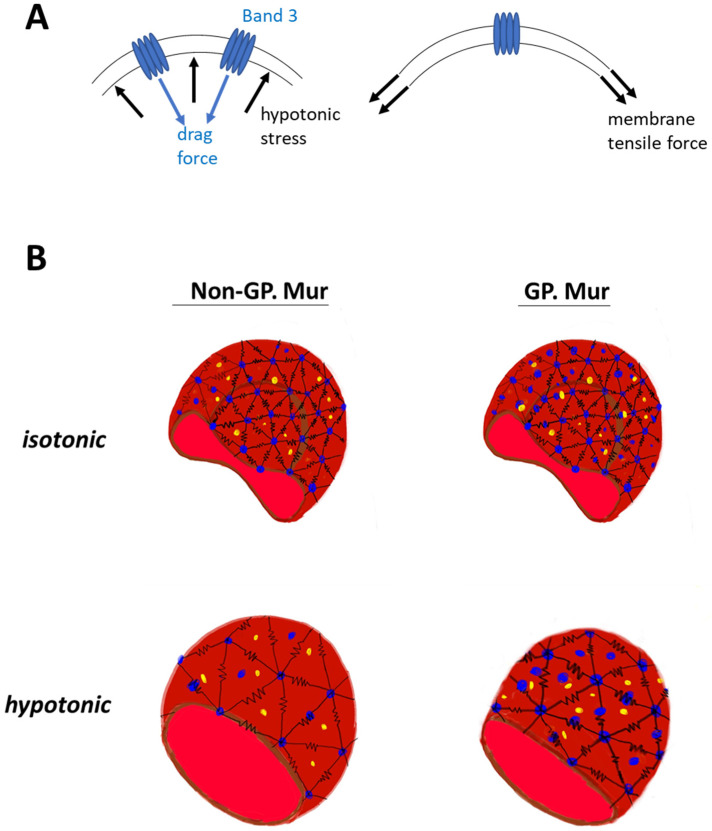
Working models: (**A**) A model illustrates how higher band 3 expression on the plasma membrane strengthens the membrane. Right: During the deformability or osmotic fragility tests, the cell membrane is extended laterally by the given membrane tension. The presence of band 3 disrupts the lateral membrane tension, as the materials that encounter the force change from lipid to protein. Left: Hypotonic stress that acts on the cell membrane and causes cell swelling is counteracted by a drag force. Because of the material differences between protein and lipid, band 3 (and its protein complexes and connected cytoskeletal network) endures a significant portion of the hypotonic stress and drag force. (**B**) GP.Mur and non-GP.Mur membrane mechanics are compared by the bead–spring model. Black springs are symbols for elastic spectrins, blue beads for tetrameric and dimeric band 3 complexes. Band 3–AQP1 complexes (blue and yellow beads) are either associated with the triangular cytoskeletal frame or independent from it (i.e., inside the triangles). Left: Non-GP.Mur normocyte. Right: GP.Mur+ normocyte. There are more band 3–AQP1 complexes on the GP.Mur+ membrane, which supports osmotic resistance to hypotonic stress.

The erythrocyte membrane structure evolved to endure forces during ~120 days of circulation. Without band 3 protein embedded in the membrane, erythrocytes would not have the biconcave shape [35]. From the mechanical viewpoint, the deformability test applies physical stress to increase lateral membrane tension (Figure 4A, right). Band 3 embedded in the cell membrane is a disruption to the lateral tension exerted onto the membrane, with its dispersed location and distinctively different material properties from the lipid bilayer. The osmotic fragility test adds hypotonic stress to the cell membrane, and a drag force is expected to act on the membrane in the opposite direction (Figure 4A, left). Cell swelling under the hypotonic stress also increases lateral membrane tension mentioned above (Figure 4A, right). If we envision the frame of a red cell as polymer chains using the bead–spring model [36,37], band 3 (blue beads in Figure 4B) endures drag force that acts on the RBC membrane. Submembranous spectrins (black springs in Figure 4B) provides elasticity to the membrane. Band 3 protein complexes and their linked cytoskeletal network strengthen the cell membrane against the forces imposed parallel and perpendicular to the cell membrane. With more band 3 molecules, GP.Mur+ RBCs can first resist stronger external force than non-GP.Mur cells, and thus were less deformable and osmotically more resistant. Second, the stronger AQP1-band 3 interaction on the GP.Mur+ cell membrane could impose additional regulation on water permeation and constrain the degree of cell swelling through band 3 interaction with ankyrin and adduction complexes (parts of the spectrin network) [19,21,22]. The importance of water channel in osmotic resistivity of RBCs was revealed when comparing GP.Mur and non-GP.Mur in this study. These two properties of band 3 complexes could thus explain why the degrees of changes in deformability and osmotic fragility appeared independent from each other (Table 3 and Table 6).

From the three factors influencing erythrocyte deformability described in the Introduction [17], neither cell volume nor intracellular viscosity (reflected by the MCHC data) significantly contributed to the deformability differences between GP.Mur+ and non-GP.Mur normocytes (Table 3 and Table 4). Rather, the slightly smaller deformability of GP.Mur+ RBCs was primarily due to membrane viscoelasticity directly or indirectly imposed by GP.Mur/higher band 3 expression. On the other hand, larger red cell volume increased chances of hypotonic burst, even after we controlled for GP.Mur phenotype and microcytotic cases in the multivariate regression model for osmotic fragility (Table 6). From these analyses, the surface area-to-volume ratio conceivably played a more important role in determining osmotic fragility than deformability.

Besides GP.Mur phenotype, membrane cholesterol also influences membrane viscoelasticity [28,29]. The rigidifying effect of membrane cholesterol on red cell deformability, however, was only observed in non-GP.Mur and not in GP.Mur+ red RBCs (Figure 3). There was also less cholesterol on the GP.Mur+ than on the non-GP.Mur erythrocyte membrane. A simplest explanation is that perhaps higher band 3 expression or/and different band 3 complex organization of GP.Mur+ RBCs could limit cholesterol absorbance onto the red cell membrane and thereby reduce the impact of membrane cholesterol on cell deformability. Reminded that red cells do not synthesize cholesterol [33], cholesterol in the red cell membrane is absorbed from cholesterol in the plasma. It remains unclear what determines cholesterol absorbance by the red cell membrane, as cholesterol levels in the serum and in the RBC membrane could not be quantitatively correlated in this study or other reports [32].

In conclusion, this study explored cellular physical distinction between red cell deformability and osmotic fragility in a large cohort comprising nearly half GP.Mur+ subjects. GP.Mur phenotype/higher band 3 expression was an important variable contributing to deformability differences and osmotic fragility differences (Table 3 and Table 6). However, it impacted the two physical properties of RBCs through distinct structural–functional roles of GP.Mur band 3 [5,11]. First, the interaction between band 3 complexes and the cytoskeleton influenced deformability, particularly in the microcytic range (Table 3 and Table 4). Second, the interaction between band 3 and AQP1 additionally influenced osmotic resistivity (Table 6). By including these factors in the modified bead–string model (Figure 4), we illustrated that the physical impact of GP.Mur in the RBC membrane was primarily on membrane viscoelasticity.

On a separate note, we unexpectedly found 25% microcytosis in GP.Mur+ women in this study (Table 1). In a separate population-based survey that is ongoing at our collaborating clinical department at Tai-Tung MMH, ~14.5% of non-GP.Mur women under 50 years old (59/406) and ~2.1% non-GP.Mur men under 50 years old (7/326) have microcytosis. From these two studies, the contribution of GP.Mur to microcytosis in women is thus unique and independent from iron-deficiency anemia (IDA) that is commonly observed among Taiwanese women at reproductive age [38]. Microcytosis is generally associated with IDA or thalassemia. Clinical evaluation of these microcytotic cases was beyond the scope and design of this study. From the RBC count of our subjects, however, all 10 GP.Mur+ women with microcytosis had <5.50 × 10^6^ RBCs per μL peripheral blood, and all three men with microcytosis had >5.50 × 10^6^ RBCs per μL (thalassemia usually accompanied with much larger compensation, i.e., >5.50 × 10^6^ RBCs per μL). This argues against more thalassemia among GP.Mur+ women. Furthermore, thalassemic RBCs are fragile, contrasting to GP.Mur+ RBCs that are more resilient to external forces (Figure 1 and Figure 2). We do not know how GP.Mur+ thalassemic RBCs would present. Future work should explore how GP.Mur or other band 3-interacting blood group components could affect red cell diagnostics.

**Limitation**: The small percentage of GP.Mur+ people remained to be a major limiting factor during subject recruitment, though the prevalence of GP.Mur special blood type in Taiwan is higher than many parts of the world.

## Figures and Tables

**Table 1:** Complete blood count (CBC) for GP.Mur+ and non-GP.Mur adult subjects in this study. The left sections included data from all subjects; the right sections compare data from subjects with and without microcytosis/hypochromia. Statistical differences between groups were estimated by two-sample *t*-test; *p* < 0.05 was deemed statistically significant; *n.s*., not significant. The number of subjects per group is indicated inside parentheses. Data are shown in mean ± SD.

CBC	All Male			All Female			Male Normocytes			Female Normocytes/Microcytes				
	Non-GP.Mur (31)	GP.Mur (28)	*p*-Value	Non-GP.Mur (46)	GP.Mur (40)	*p*-Value	Non-GP.Mur Normocytes (30)	GP.Mur Normocytes (26)	*p*-Value	Non-GP.Mur Normocytes (46)	GP.Mur Normocytes (30)	GP.Mur Microcytes (10)	*p*-Value (non-GP.Mur vs. GP.Mur Normocytes)	*p*-Value (GP.Mur+ Normocytes vs. Microcytes)
RBC (10^6^/µL)	5.1 ± 0.3	5.0 ± 0.4	*n.s.*	4.4 ± 0.5	4.6 ± 0.4	*n.s.*	5.0 ± 0.3	5.0 ± 0.4	*n.s.*	4.4 ± 0.5	4.5 ± 0.3	5.0 ± 0.4	*n.s.*	*p* < 0.01
HgB (g/dL)	15.1 ± 1.0	14.6 ± 1.1	*n.s.*	13.0 ± 0.9	12.3 ± 1.7	*p* < 0.05	15.2 ± 0.9	14.7 ± 1.0	*n.s.*	13.0 ± 0.9	13.0 ± 0.9	10.3 ± 1.8	*n.s.*	*p* < 0.01
HCT (%)	45.7 ± 2.6	44.6 ± 2.7	*n.s.*	39.9 ± 2.6	38.3 ± 4.4	*n.s.*	45.9 ± 2.5	44.8 ± 2.7	*n.s.*	39.9 ± 2.6	40.0 ± 2.7	33.1 ± 4.8	*n.s.*	*p* < 0.01
MCV (fL)	90.7 ± 4.8	90.1 ± 6.1	*n.s.*	90.2 ± 6.1	83.8 ± 11.8	*p* < 0.01	91.2 ± 3.7	90.6 ± 4.4	*n.s.*	90.2 ± 6.1	89.4 ± 5.3	66.8 ± 9.2	*n.s.*	*p* < 0.001
MCH (pg/cell)	30.0 ± 1.9	29.5 ± 2.5	*n.s.*	29.5 ± 2.5	26.9 ± 4.4	*p* < 0.01	30.2 ± 1.4	29.8 ± 1.9	*n.s.*	29.5 ± 2.5	29.1 ± 2.0	20.7 ± 3.6	*n.s.*	*p* < 0.001
MCHC (g/dL)	33.0 ± 0.7	32.7 ± 0.9	*n.s.*	32.7 ± 0.8	32.1 ± 1.0	*p* < 0.01	33.1 ± 0.6	32.8 ± 0.8	*n.s.*	32.7 ± 0.8	32.5 ± 0.6	30.9 ± 1.2	*n.s.*	*p* < 0.01
RDW (%)	13.5 ± 0.7	14.0 ± 1.2	*p < 0.05*	13.9 ± 1.1	15.5 ± 3.2	*p* < 0.01	13.5 ± 0.7	13.8 ± 0.7	*n.s.*	13.9 ± 1.1	14.2 ± 1.6	19.4 ± 3.5	*n.s.*	*p* < 0.01
RDW-SD (fL)	42.1 ± 3.4	43.1 ± 4.8	*n.s.*	43.3 ± 3.3	43.6 ± 4.7	*n.s.*	42.3 ± 3.2	42.6 ± 2.6	*n.s.*	43.4 ± 3.3	43.5 ± 5.0	44.0 ± 3.8	*n.s.*	*n.s.*
% microcytosis	3.2% (1/31)	7.1% (2/28)		0% (0/46)	25% (10/40)		0%	0%		0%	0%	100%		

**Table 2:** The RBC deformability test results and the osmolarity fragility test results for GP.Mur-positive and GP.Mur-negative subjects.

Deformability Test	Non-GP.Mur Normocytes	GP.Mur Normocytes	GP.Mur Microcytes	*p*-Value (Non-GP.Mur vs. GP.Mur+ Normocytes)	*p*-Value (GP.Mur+ Normocytes vs. Microcytes)
EI at 1 pa	0.183 ± 0.020	0.183 ± 0.016	0.133 ± 0.029	*n.s.*	*p* < 0.001
EI at 3 pa	0.326 ± 0.020	0.323 ± 0.019	0.270 ± 0.047	*n.s.*	*p* < 0.01
EI at 7 pa	0.421 ± 0.022	0.414 ± 0.023	0.359 ± 0.050	*n.s.*	*p* < 0.001
EI at 10 pa	0.455 ± 0.017	0.447 ± 0.020	0.409 ± 0.034	*n.s.*	*p* < 0.01
EI at 20 pa	0.500 ± 0.016	0.490 ± 0.022	0.444 ± 0.037	*p* < 0.001	*p* < 0.001
EI_max_	0.550 ± 0.017	0.539 ± 0.022	0.492 ± 0.039	*p* < 0.001	*p* < 0.001
osmolarity fragility test					
%NaCl at which hemolysis began	0.486 ± 0.043	0.464 ± 0.038	0.423 ± 0.041	*p* < 0.05	*p* < 0.01
%NaCl at which hemolysis terminated	0.288 ± 0.067	0.218 ± 0.070	0.173 ± 0.065	*p* < 0.001	*n.s.*

**Table 3:** In this multivariate regression analysis for EI_max_, gender, GP.Mur phenotype, microcytosis, and %NaCl at which hemolysis began and ended were the independent variables. *R*, or the multiple correlation coefficient for this model, was 0.625, suggesting a good level of prediction of EI_max_ using these independent variables. β is the unstandardized coefficient which indicates the magnitude of the effect of an independent variable when other independent variables are controlled. Standardized β suggests the relative contribution of all the independent variables in this regression model. The *p*-value indicates whether an independent variable indeed contributes significantly to the model for EI_max_; *** p < 0.001; * p < 0.05; *n.s*., not significant. This model showed that (1) EI_max_ (the main indicator for RBC deformability) was independent from osmotic fragility and (2) RBC deformability was significantly influenced by individual GP.Mur phenotype and microcytosis/hypochromia.

EI_max_	β	SE β	Standardized Coefficients β	*p*-Value	95% CI for β	
					Lower	Upper
Model (R = 0.625)				***		
constant	0.531	0.027		***	−0.478	0.584
gender	−0.002	0.005	−0.041	*n.s.*	−0.011	0.007
GP.Mur	−0.01	0.005	−0.191	*	−0.021	0.000
microcytosis	−0.048	−0.009	−0.502	***	−0.065	−0.031
%NaCl at which hemolysis began	0.040	0.058	0.067	*n.s.*	−0.075	0.155
%NaCl at which hemolysis ended	0.006	0.036	0.017	*n.s.*	−0.065	−0.031

**Table 4:** Three multivariate regression models of EI_max_ for the non-GP.Mur normocytic, GP.Mur normocytic, and GP.Mur microcytic groups. The models for the non-GP.Mur normocytic and GP.Mur microcytic groups could be explained by the two independent variables, membrane cholesterol and MCV, with significance, but the model for the GP.Mur normocytic group could not. These three models revealed distinctly different contribution of membrane cholesterol contents and MCV to deformability with respect to GP.Mur type and microcytosis. *** *p* < 0.001; ** *p* < 0.01; * *p* < 0.05; *n.s*., not significant.

EI_max_	β	SE β	Standardized Coefficients β	*p*-Value	95% CI for β	
					Lower	Upper
non-GP.Mur normocyte model (R = 0.334)				***		
constant	0.499	0.037		***	0.425	0.572
membrane cholesterol	−0.003	0.001	−0.285	*	−0.005	0.000
MCV	0.001	0.000	0.208	*n.s.*	0.000	0.001
GP.Mur normocyte model (R = 0.082)				*n.s.*		
constant	0.564	0.061		***	0.441	0.687
membrane cholesterol	−0.002	0.004	−0.072	*n.s.*	−0.010	0.006
MCV	0.000	0.001	−0.045	*n.s.*	−0.002	0.001
GP.Mur microcyte model (R = 0.877)				**		
constant	0.276	0.048		***	0.167	0.385
membrane cholesterol	0.000	0.006	0.008	*n.s.*	−0.013	0.013
MCV	0.003	0.001	0.879	***	0.002	0.004

**Table 5:** The levels of serum cholesterol and membrane cholesterol in GP.Mur-positive and GP.Mur-negative adult subjects. ** *p* < 0.01; * *p* < 0.05; *n.s*., not significant.

All Subjects	Non-GP.Mur	GP.Mur	*p*-Value
serum cholesterol (mg/dL)	138.7 ± 27.9	145.2 ± 37.6	n.s.
membrane cholesterol (μM)	3.66 ± 1.76	3.03 ± 0.86	* *p* < 0.05
normocytic subjects	non-GP.Mur	GP.Mur	*p*-value
serum cholesterol (mg/dL)	136.7 ± 26.2	150.0 ± 35.7	n.s.
membrane cholesterol (μM)	3.65 ± 1.76	2.98 ± 0.80	** *p* < 0.01

**Table 6:** A multivariate regression model for osmotic fragility included GP.Mur phenotype, microcytosis, MCV, RDW, MCHC, and EI_max_, as the independent variables. Osmotic fragility was represented by %NaCl at which hemolysis terminated. This model with R ~ 0.629 presented a good level of prediction for osmotic fragility, with two significant contributors—MCV and GP.Mur phenotype (*** p < 0.001; ** p < 0.01). The former factor MCV promoted osmotic fragility of red cells (positive standardized β). The latter factor GP.Mur reduced osmotic fragility (negative standardized β). This model also showed that osmotic fragility was independent from other CBC parameters (MCHC and RDW), microcytosis, or EI_max_ (*p* > 0.05). *n.s*., not significant.

%NaCl at Which Hemolysis Terminated (Osmotic Fragility)	β	SE β	Standardized Coefficients β	*p*-Value	95% CI for β	
					Lower	Upper
model (R = 0.629)				***		
constant	0.610	0.371		*n.s.*	−0.127	1.347
GP.Mur	−0.072	0.014	−0.449	***	−0.10	−0.043
microcytosis	0.002	0.036	0.009	*n.s.*	−0.068	0.073
MCV	0.004	0.001	0.392	**	0.001	0.006
RDW	−0.007	0.006	−0.171	*n.s.*	−0.018	0.004
MCHC	−0.012	0.011	−0.136	*n.s.*	−0.034	0.010
EI_max_	−0.290	0.333	−0.098	*n.s.*	−0.952	0.373

## Data Availability

Data available on reasonable request due to ethical restrictions by MMH-IRB.
